# Uncertainty and hotspots in 21st century projections of agricultural drought from CMIP5 models

**DOI:** 10.1038/s41598-019-41196-z

**Published:** 2019-03-20

**Authors:** Junyu Lu, Gregory J. Carbone, John M. Grego

**Affiliations:** 1 Department of Geography, University of South Carolina, Columbia, South Carolina 29208 United States; 2 Department of Statistics, University of South Carolina, Columbia, South Carolina 29208 United States

## Abstract

Future climate changes could alter hydrometeorological patterns and change the nature of droughts at global to regional scales. However, there are considerable uncertainties in future drought projections. Here, we focus on agricultural drought by analyzing surface soil moisture outputs from CMIP5 multi-model ensembles (MMEs) under RCP2.6, RCP4.5, RCP6.0, and RCP8.5 scenarios. First, the annual mean soil moisture by the end of the 21st century shows statistically significant large-scale drying and limited areas of wetting for all scenarios, with stronger drying as the strength of radiative forcing increases. Second, the MME mean spatial extent of severe drought is projected to increase for all regions and all future RCP scenarios, and most notably in Central America (CAM), Europe and Mediterranean (EUM), Tropical South America (TSA), and South Africa (SAF). Third, the model uncertainty presents the largest source of uncertainty (over 80%) across the entire 21st century among the three sources of uncertainty: internal variability, model uncertainty, and scenario uncertainty. Finally, we find that the spatial pattern and magnitude of annual and seasonal signal to noise (S/N) in soil moisture anomalies do not change significantly by lead time, indicating that the spreads of uncertainties become larger as the signals become stronger.

## Introduction

Future drought risks could be exacerbated by spatiotemporal changes in hydrometeorological variables due to climate change^1–3^. Warming associated with climate change accelerates land surface drying, enhances evapotranspiration, and increases the potential incidence and severity of droughts^2^. Changes in the frequency, intensity, and duration of droughts would have significant impacts on water management, natural resources, agriculture, aquatic ecosystems, and socioeconomic sectors. In the context of climate change, it is important for decision makers to understand how drought conditions might change at the regional scale in order to plan adequate adaptation and mitigation strategies^4^.

Drought is a complex multivariate phenomenon caused by the interaction of atmospheric, hydrological, and biogeophysical processes. There are considerable uncertainties in evaluating drought trends both in the instrumental record and future drought projections. For example, the IPCC Fourth Assessment Report (AR4)^2^ concluded that, since the 1970s, more intense and longer droughts have been observed over wider areas, particularly in the tropics and subtropics, which are linked with higher temperatures and decreased precipitation. However, the IPCC Fifth Assessment Report (AR5)^5^ indicated that the global increasing trends in drought since 1970 were no longer supported. Recent evidence has yielded conflicting results on drought changes^6,7^. Such phenomena demonstrate the challenges and uncertainties in quantitatively detecting long-term changes of this complex phenomenon. In addition to uncertainties with respect to past observations, there are even more considerable uncertainties associated with future drought projection centering on a different set of factors including inherent climate variability, model errors, and uncertainty in future radiative forcing.

Prior works investigating potential changes in drought have revealed that different drought indices can produce different results due to different variables involved in calculating the drought indices from the general circulation models (GCMs)^8–12^. The drought indices including temperature and evapotranspiration effects, e.g., Palmer Drought Severity Index (PDSI)^13^, Standardized Precipitation Evapotranspiration Index (SPEI)^14^, Supply-Demand Drought Index (SDDI)^15^, etc., usually project stronger drought risks than the drought indices using only precipitation or runoff, e.g., Standardized Precipitation Index (SPI)^16^, Standardized Runoff Index (SRI)^17^, etc^8–12^.

Droughts involve a wide range of related variables. Different types of drought highlight different variables of interest, for example, meteorological droughts highlight precipitation, agricultural droughts highlight soil moisture, and hydrological droughts highlight streamflow/runoff ^18,19^. In this study, we focus on agricultural drought. Agricultural droughts reduce soil-water availability, affect crop production and yield, and pose threats to food security and livestock industries^1,20^. Soil moisture is an important indicator for agricultural drought since it can reflect the total effects of precipitation and evapotranspiration, represent the status of agriculture, and determine the available water supply for healthy plant growth^18,21,22^. Modeling soil moisture changes is much more complicated than precipitation and temperature. Future soil moisture changes depend on the total interaction of temperature and precipitation, the complex surface hydrological process, as well as other factors, such as wind speed, vegetation, land use/cover change, and atmospheric CO_2_ (influence plant stomatal conductance and hence plant transpiration)^23^. Thus, we use soil moisture as an integrative variable to reflect the change in agricultural drought risks.

Prior studies have assessed and quantified the uncertainty associated with primary climate variables like surface air temperature^24,25^ and precipitation^26,27^. There also have been several efforts^28,29^ to evaluate the performance of GCMs to simulate the soil moisture using in situ or satellite observations and several attempts^23,30,31^ to project future drought conditions using soil moisture data from GCMs. However, fewer studies have assessed and quantified the sources of uncertainties in projecting future agricultural drought conditions. AR5 stated that the regional to global-scale projections of drought conditions remain relatively uncertain compared to other aspects of the water cycle^5^. Understanding and modeling uncertainties and hotspots in drought projection are of great importance in natural resources and water resources planning management. Quantifying and partitioning uncertainty associated with drought is also very important for decision makers to understand the scope and direction for narrowing the uncertainty through investment in climate science^24^.

Here, we use all available GCMs providing surface soil moisture outputs under the fifth phase of the Coupled Model Intercomparison Project (CMIP5), which enable us to capture model uncertainty in the representation of climate sensitivity and climate process. We use all available representative concentration pathway (RCP) scenarios: RCP 2.6, RCP 4.5, RCP 6.0, and RCP 8.5, which enable us to understand the uncertainty originating from unknown future greenhouse gas emissions and radiative forcing. We focus on agricultural drought and use soil moisture as an important indicator for agricultural drought. We analyze the raw GCMs outputs for surface soil moisture, instead of computing drought indices from related variables, which may introduce additional uncertainty. First, we investigate the multi-model ensemble (MME) annual and seasonal percentage change of surface soil moisture and evaluate the statistical significance of change using a Wilcoxon signed-rank test for each grid by controlling the false discovery rate (FDR) at a significance level of 0.05. Second, we calculate the duration, frequency, severity, and spatial extent of severe agricultural drought and analyze the spatial-temporal change of those drought characteristics. Third, we quantify and partition the three sources of uncertainty associated with these drought projections: internal variability, model uncertainty, and scenario uncertainty. Finally, we examine the spatiotemporal variability of annual and seasonal signal to noise (S/N) change in soil moisture anomalies across the globe and for different lead times to measure the magnitude of the expected change compared with the uncertainty in the projection.

## Results

### Global multi-model mean surface soil moisture change

**Figure 1:**
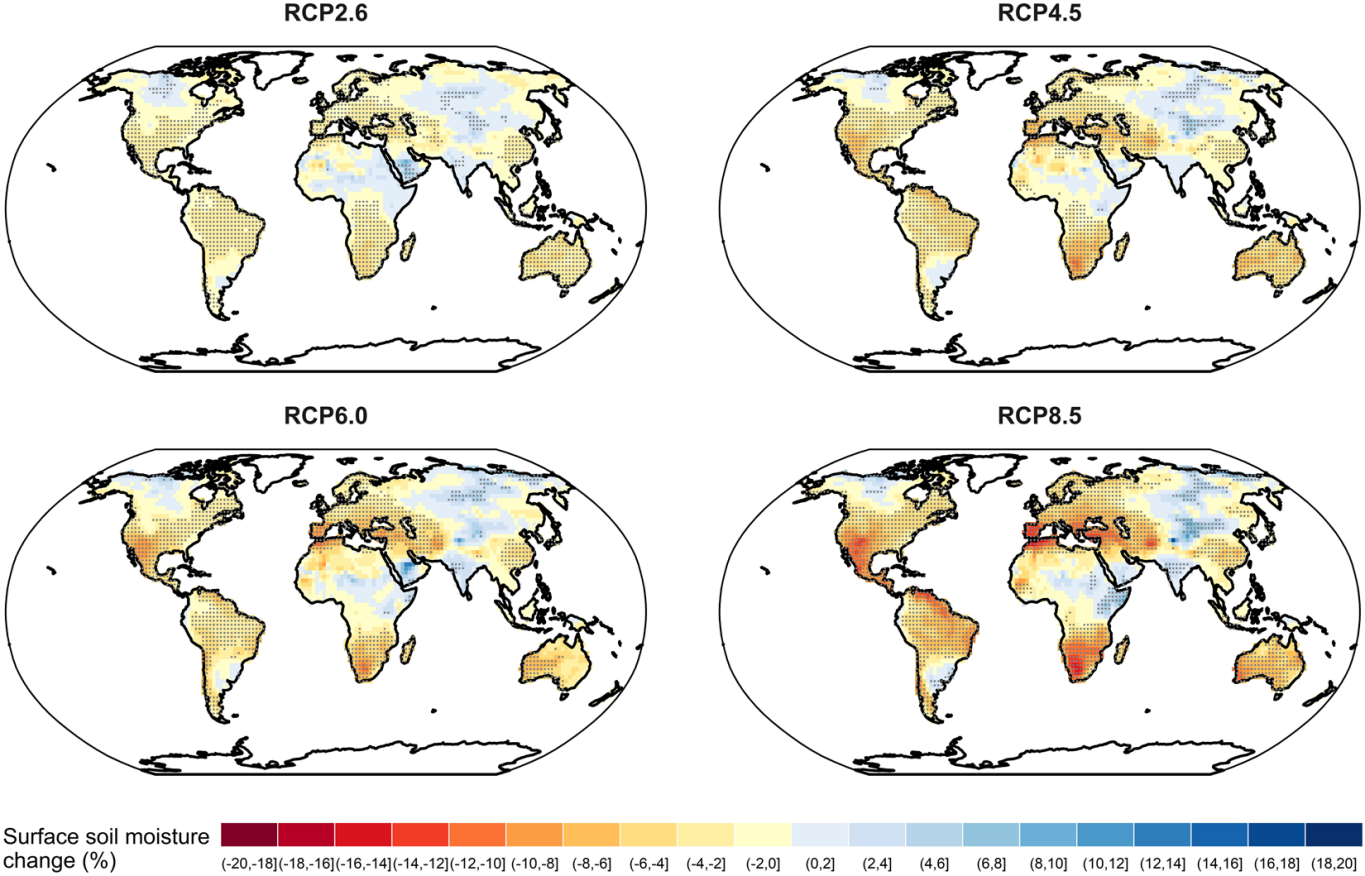
Global multi-model mean percentage change in annual mean surface soil moisture for the period of 2071–2100 (RCP forcing) relative to 1976–2005 (historical forcing) based on CMIP5 multi-model ensembles (MMEs) under four scenarios: RCP2.6, RCP4.5, RCP6.0, and RCP8.5. The grids with stippling indicate statistical significance using the non-parametric Wilcoxon signed-rank test by controlling the false discovery rate (FDR) at a significance level of 0.05, i.e., there is a strong evidence that the distribution of annual mean surface soil moisture from different GCMs for period of 2071–2100 and the period of 1976–2005 is not the same for those grid cells. The results are based on all available models for each RCP scenario and the corresponding models in the historical forcing.

We investigate future agricultural drought change by calculating the multi-model mean percentage change in annual/seasonal mean surface soil moisture for the period of 2071–2100 (RCP forcing) relative to 1976–2005 (historical forcing) for each emission scenario (Fig. 1). Percentage change is calculated because the magnitude of surface soil moisture varies by model; a 30-year period is chosen to sufficiently filter out interannual variability, but maintain multi-decadal variability. We also evaluate the statistical significance of change using a Wilcoxon signed-rank test, which is a nonparametric distribution-free paired-sample hypothesis testing procedure. The Wilcoxon signed-rank test is usually used as an alternative to paired two-sample student t-test when the assumption of normality is violated. Here, 9746 out of 29376 (33.18%) Shapiro-Wilk normality tests on a per-pixel basis reject the null hypothesis that the annual mean surface soil moisture from different GCMs for the period of 1976–2005 or the period of 2071–2100 are normally distributed at a significance level of 0.05. Thus, we perform a Wilcoxon signed-rank test (two-tailed) for each grid and each scenario by testing the null hypothesis that the distribution of the annual/seasonal mean surface soil moisture for different GCMs for the period of 1976–2005 (historical forcing) and the period of 2071–2100 (RCP forcing) are the same. A paired two-sample hypothesis test is used to control sources of variability in which the annual/seasonal mean soil moisture for the two 30-year periods from the same GCM is a matched-pair sample. An independent two-sample test is not appropriate in such cases. Moreover, since we perform multiple comparisons using the Wilcoxon signed-rank test and calculate the p-values for more than 3000 grids for each scenario, we control for the false discovery rate (FDR) (i.e., the expected proportion of false discoveries among the total number of discoveries) for multiple comparisons at a significance level of 0.05 and adjust the p-value for each grid cell following the method of Benjamini and Hochberg^32^ (Fig. 1).

The annual mean surface soil moisture by the end of the 21st century shows statistically significant large-scale drying over most of Australia, South America, North America, southern Africa, Europe and Mediterranean, and east Asia, and statistically significant wetting in limited areas of east Africa, south Asia, and central Asia (Fig. 1). The overall spatial patterns of drying and wetting are generally consistent across the four RCP scenarios, with stronger drying as forcing increases (Fig. 1). The soil moisture drying in the Mediterranean, southwestern USA, northeast South America, and southern Africa is associated with projected widening of the Hadley Circulation that shifts downwelling and inhibits precipitation in these regions and globally increased temperature and evapotranspiration^5^.

The changing signal is more pronounced in winter and summer than annually because of compensating effects during the whole year (Fig. 1, Supplementary Figs S1 and S2). We have also detected a strong seasonality in many regions in the mid- and high-latitudes of the North Hemisphere, with wetting in the winter and drying in the summer (Supplementary Figs S1 and S2) which is most likely due to increased temperature and evapotranspiration, increased precipitation throughout whole year, and earlier melting of ice and snow.

### Global multi-model drought characteristics change

**Figure 2:**
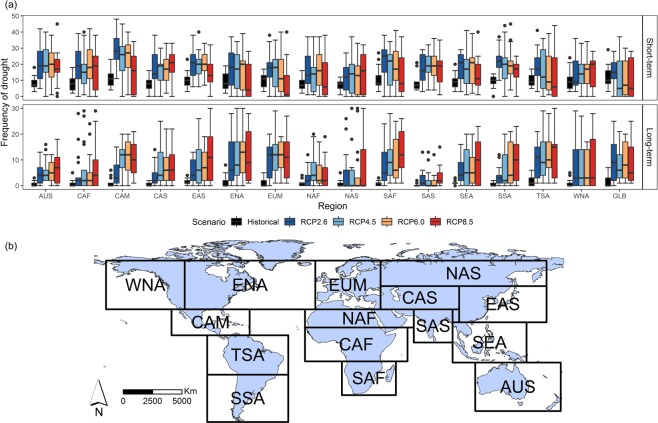
(**a**) Boxplots of global mean frequency of short-term drought (longer than or equal to 2 months and less than 6 months, see methods), frequency of long-term drought (longer than or equal to 6 months, see methods) for the period of 1976–2005 (historical forcing) and 2071–2100 (RCP forcing) under four emission scenarios: RCP2.6, RCP4.5, RCP6.0, and RCP8.5. The range of the variation for each period is based on CMIP5 MMEs. (**b**) 15 regions defined in IPCC^5^: Western North America (WNA), Eastern North America (ENA), Central America (CAM), Tropical South America (TSA), Southern South America (SSA), Europe and Mediterranean (EUM), North Africa (NAF), Central Africa (CAF), South Africa (SAF), North Asia (NAS), Central Asia (CAS), East Asia (EAS), South Asia (SAS), Southeast Asia (SEA) and Australia (AUS). The GLB stands for Globe.

We calculate the drought characteristics: frequency of short-term drought (longer than or equal to 2 months and less than 6 months) and frequency of long-term drought (longer than or equal to 6 months) for the 30-year period of 1976–2005 (historical forcing) and 2071–2100 (RCP forcing) for each region (Fig. 2(a)). The multi-model median (shown in the boxplots) frequency of short-term drought is projected to increase by the end of the 21st century for most regions and for most RCP scenarios (Fig. 2(a)). In most cases, the increase in the frequency of short-term drought is higher for RCP2.6 than RCP8.5. There are several cases where the frequency of short-term drought is projected to decrease for the highest radiative forcing RCP8.5 compared to the historical period, such as Eastern North America (ENA), Europe and Mediterranean (EUM), South Africa (SAF), Tropical South America (TSA), and the global. This is because, in the RCP8.5 scenario, sequences of consecutive dry months are more likely, thus increasing the frequency of long-term drought and decreasing the frequency of short-term drought. Figure 2(a) shows that the median frequency of long-term drought is projected to increase in most regions, with the greatest increase in EUM, TSA, Central America (CAM), ENA, and SAF, and the smallest increase in North Asia (NAS), South Asia (SAS), Central Africa (CAF), and North Africa (NAF). The highest radiative forcing shows the greatest increase in the long-term drought in most regions. However, as the boxplot show, the MMEs have a range that is much larger than the change (Fig. 2(a)).

**Figure 3:**
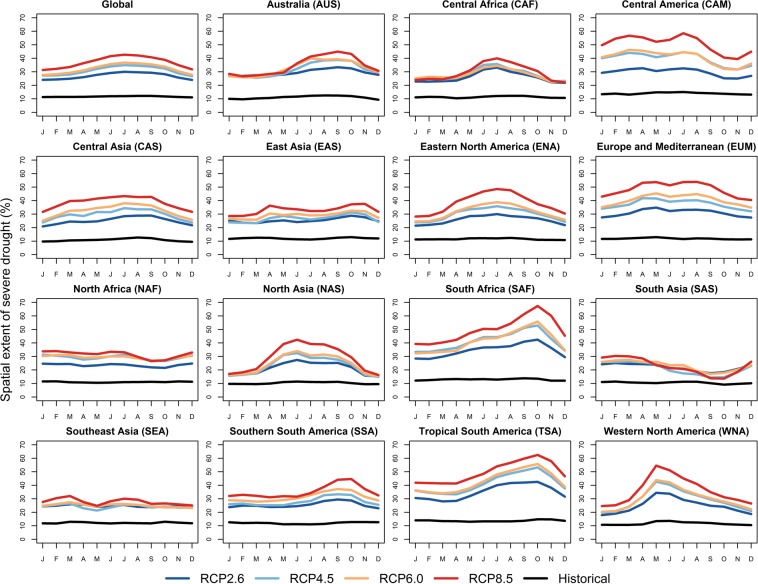
Global and regional multi-model ensemble mean, 30-year mean of the monthly spatial extent of severe drought for the period of 1976–2005 in historical forcing and 2071–2100 in RCP forcing under four emission scenarios: RCP2.6, RCP4.5, RCP6.0, and RCP8.5.

The global multi-model ensemble mean in the spatial extent of severe drought is projected to increase from approximately 11% for the period of 1976–2005 to 27% under RCP2.6, 29% under RCP4.5, 32% under RCP6.0, and 33% under RCP 8.5 for the period of 2071–2100 (Fig. 3). Figure 3 shows the regional seasonal patterns of future drought projections in the RCP forcing compared with the historical forcing. The multi-model mean spatial extents of severe drought are projected to increase for all regions and all future RCP scenarios, with progressively larger spatial extent of severe drought as the strength of radiative forcing increases (RCP8.5 > RCP6.0 > RCP4.5 > RCP2.6) in most cases. The increase in the spatial extent of drought tends to be larger in warmer seasons than cooler seasons in most regions. In southern hemisphere (TSA, SSA, AUS, and SAF), the largest increase in spatial extent occurs predominantly in the Austral Spring. The increase in the spatial extent of soil moisture deficit in high latitude regions (e.g. NAS) tends to be concentrated in the warmer season and diminished in the cooler season. The seasonal disproportionate change of soil moisture deficit is mainly because of changes in snow and ice. During the cooler season, the temperature increase tends to reduce the snow cover, increase the ratio of rainfall to snowfall, and drive earlier spring melting which limits soil moisture deficit, while during the warmer season, earlier spring melting coupled with higher evapotranspiration strengthen the soil moisture deficit^23^. This mechanism leads to the seasonal disproportionate change in the spatial extent of drought in warmer months compared with the cooler months in high latitude regions.

CAM and EUM show the largest spread across emission scenarios, i.e., these regions respond very differently to different radiative forcing compared with other regions (Fig. 3). For those regions, the highest radiative forcing (RCP8.5) creates a much greater spatial extent of drought than the lowest radiative forcing (RCP2.6). By contrast, NAF, SAS, SEA, and CAF show the smallest spread resulting from different emission scenarios (Fig. 3), i.e., the spatial extent of drought in these regions is relatively insensitive to the differences in radiative forcing compared with other regions.

**Figure 4:**
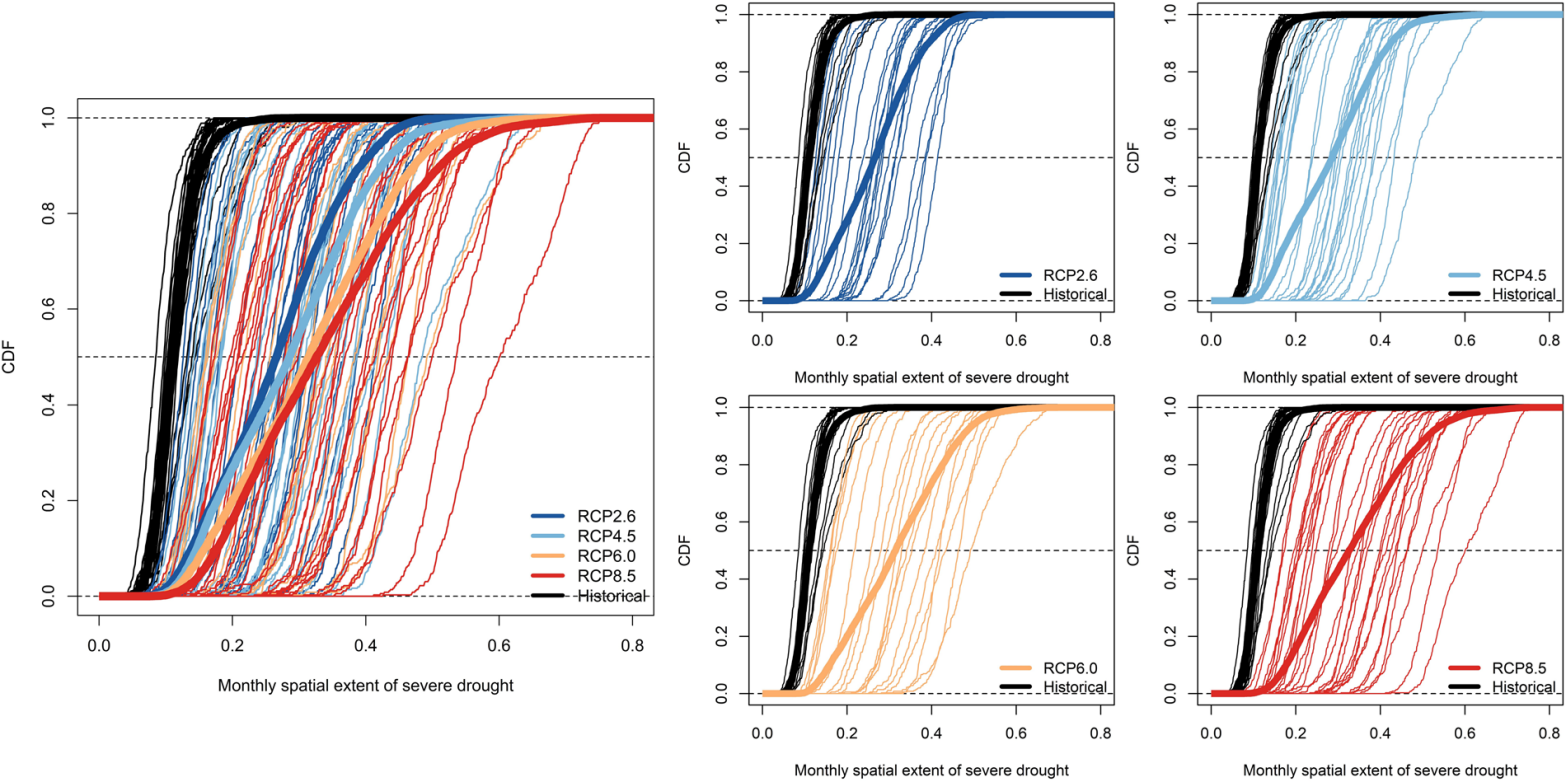
Empirical cumulative distribution functions (CDFs) of the global monthly spatial extent of severe drought for the period of 1976–2005 (360 months) in historical forcing and 2071–2100 (360 months) in RCP forcing under four emission scenarios: RCP2.6, RCP4.5, RCP6.0, and RCP8.5. The thin lines indicate the CDFs for individual GCMs and the thick lines indicate the CDFs for all GCMs for each scenario.

We fit empirical CDFs of the global monthly spatial extent of drought for each individual model for the 360-month in the period of 1976–2005 (historical forcing) and the 360-month in the period of 2071–2100 (RCP forcing) to investigate both the mean change and temporal variability (inter-month variability) change (Fig. 4). Most of the GCMs project increases in the spatial extent of severe drought, in which the RCP8.5 shows the largest increase. The MMEs under RCP forcing show very large inter-model uncertainty, in which RCP8.5 shows the widest range of projections, while RCP2.6 shows the narrowest range of projections, indicating that the projection uncertainty increases as the radiative forcing increases. This is true when the four RCP scenarios contain the same set of GCMs (not shown here). Furthermore, for each individual GCM projection under RCP forcing, in most cases, the CDFs, especially under RCP8.5, are flatter when compared with the CDFs using historical forcing, indicating greater temporal (inter-month) variability in the spatial extent of severe drought, i.e., more widespread drought and more extreme drought events.

**Figure 5:**
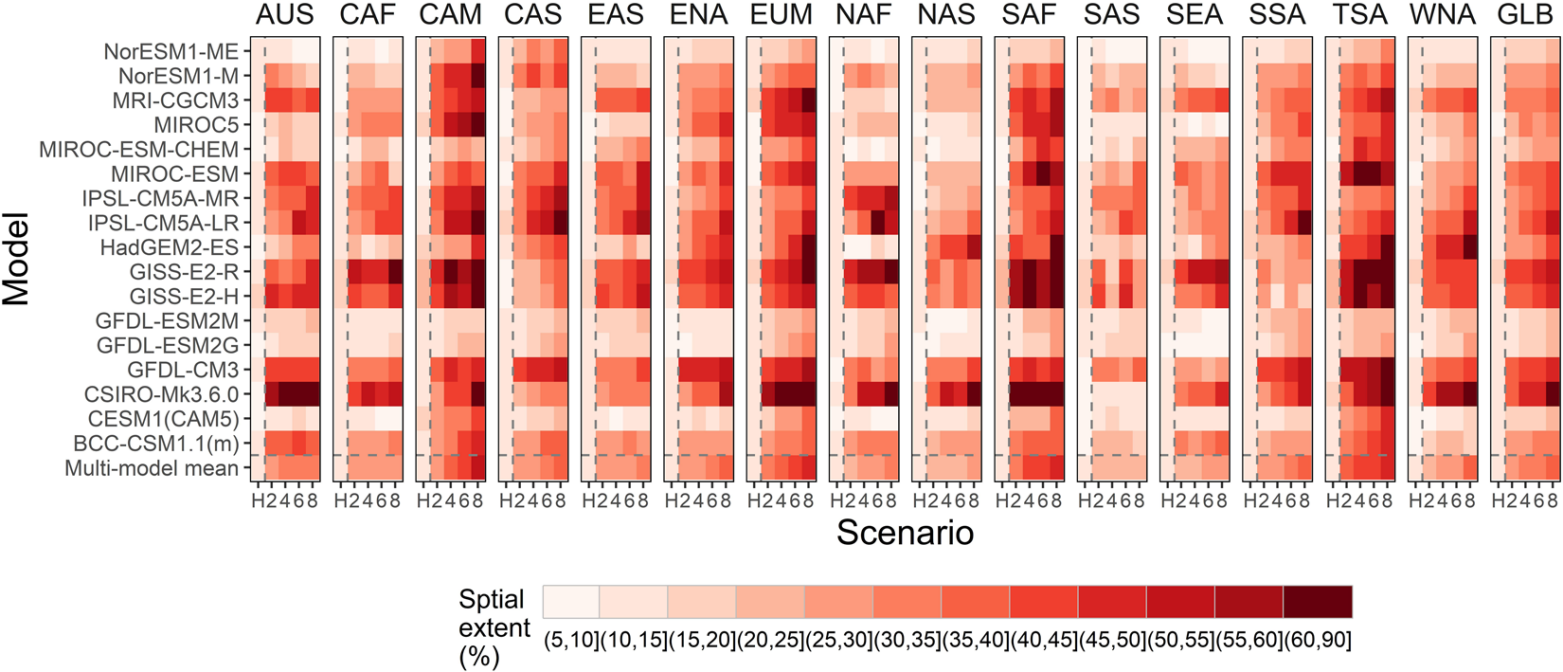
Mean spatial extent of severe drought for the period of 1976–2005 in historical forcing and for the period of 2071–2100 in RCP forcing under four emission scenarios: RCP2.6, RCP4.5, RCP6.0, and RCP8.5. H, 2, 4, and 8 in the x-axis represent historical, RCP2.6, RCP4.5, RCP6.0, and RCP8.5 scenario respectively. The columns are globe (GLB) and 15 regions.

We calculate 30-year mean of the spatial extent of severe drought for the globe and for 15 regions for the period of 1976–2005 (historical forcing) and the period of 2071–2100 (RCP forcing) (Fig. 5). The model results from the same institution are similar and highly correlated (e.g. GISS-E2-R and GISS-E2-H, IPSL-CM5A-MR and IPSL-CM5A-LR, GFDL-ESM2M and GFDL-ESM2G) and those climate models developed by the same institution and sharing model components might have shared biases. The multi-model and multi-scenario mean spatial extent of CAM, EUM, TSA, and SAF are projected to increase the most, while the mean spatial extent of SAS, SEA, NAS, and CAF are projected to increase the least. We find that the variations of the spatial extent of drought across the 17 models (standard deviation: 10.4, unit: %) are much larger than the variations across the 4 RCP scenarios (standard deviation: 4.2, unit: %), i.e., the model spread is much larger than the scenario spread. Also, we find that the variations across the 17 models (standard deviation: 10.4, unit: %) are also larger than the variations across those 15 regions (standard deviation: 6.4, unit: %). Thus, the model difference is a significant contribution to the uncertainty of the future drought projections.

### Uncertainties in projection of global mean severe drought

**Figure 6:**
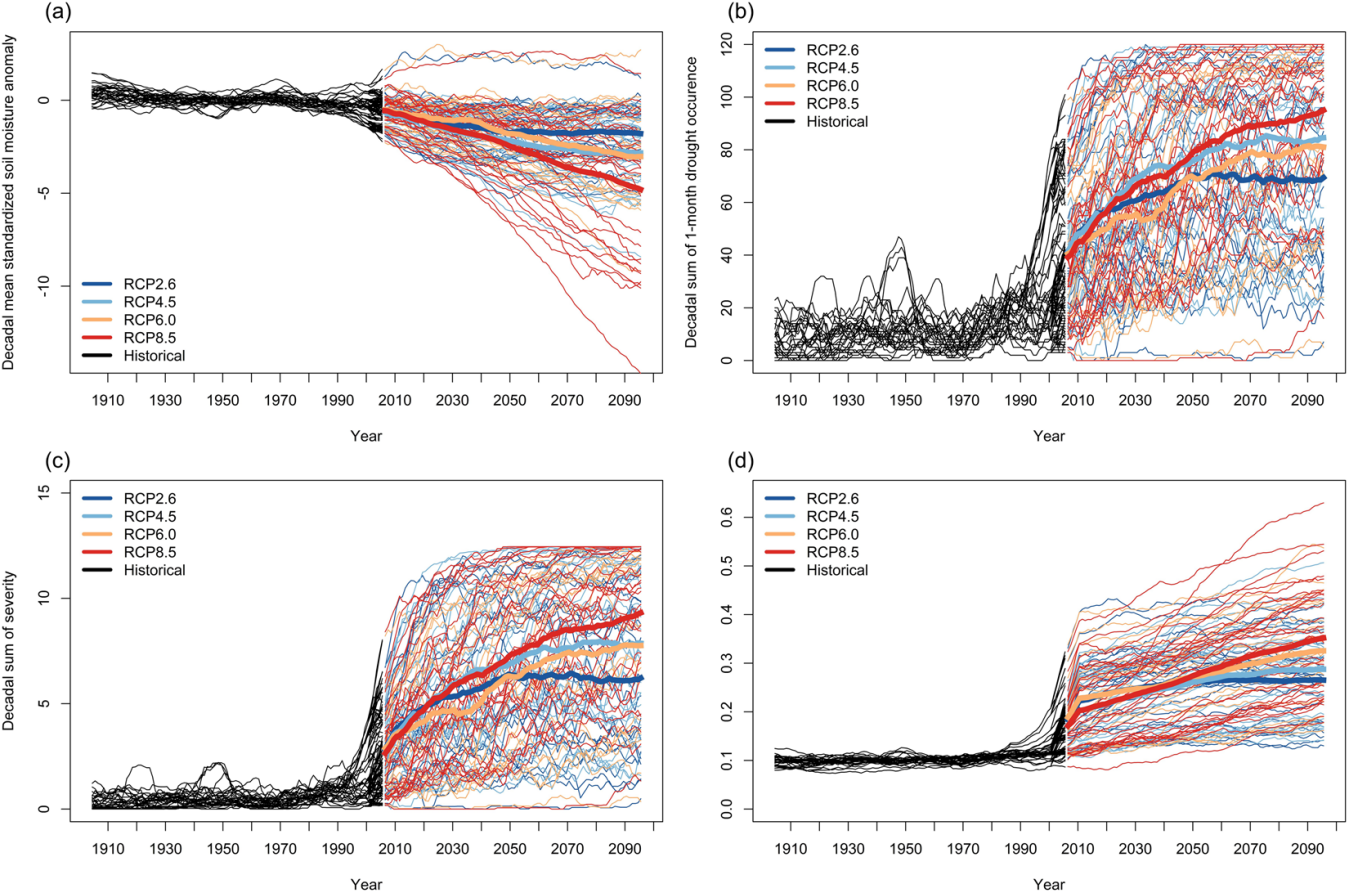
Global (**a**) decadal mean standardized soil moisture anomalies; (**b**) decadal sum of 1-month drought occurrence; (**c**) decadal sum of drought severity; (**d**) decadal mean spatial extent of drought from CMIP5 MMEs under four RCP scenarios: RCP2.6, RCP4.5, RCP6.0, and RCP8.5 from 1900 to 2100 (the thin line represents individual GCM and the thick line represents multi-model mean for each scenario). The fluctuations (“wiggles”) superimposed on the long-term trends in each projection approximate the internal variability in climate; the spread of the thin lines in the same color represents the model uncertainty for a particular scenario (e.g. red color for RCP8.5); the spread of the four thick colored lines represents the scenario uncertainty.

Figure 6(a) shows time series of global decadal mean standardized soil moisture anomalies. We perform a Mann-Kendall trend test for the time series for each model and each scenario and we found that only model FGOALS-s2 in RCP2.6, RCP6.0, and RCP8.5 show a significantly positive trend, only model CESM1(CAM5) in RCP6.0 does not show a significant trend, and all other models in all four RCP scenarios show a significantly negative trend at a significance level of 0.05. Soil moisture is projected to decrease in the twenty-first century, with the strongest drying associated with the highest emissions scenario. Figure 6(b) shows time series of global decadal sum of 1-month drought occurrence, representing the month counts in a 10-year moving window when soil moisture values fall below the 10th percentile of the historical simulation (1900–2005). Figure 6(c) shows time series of global decadal sum of severity, representing the sum of severity for all drought events in a 10-year moving window. The time series of decadal sum of 1-month drought occurrence and sum of severity show similar results, both are projected to increase over the 21st century with the highest increase for RCP8.5, and the lowest increase for RCP6.0 before mid-century and for RCP2.6 after mid-century. Figure 6(d) shows time series of the global decadal mean spatial extent of severe drought, representing the global decadal mean percentage of areas experiencing severe drought conditions. The multi-model mean is projected to increase from approximately 10% during the 20th century to 26.5% (RCP2.6) and 35.2% (RCP8.5) by the end of the 21st century. Collectively, Fig. 6 shows that the spread of different models in response to the same radiative forcing (the spread of thin lines of the same color) is much larger than the spread of the different responses depending on the radiative forcing (RCP) (the spread of four thick lines) for the 21st century.

**Figure 7:**
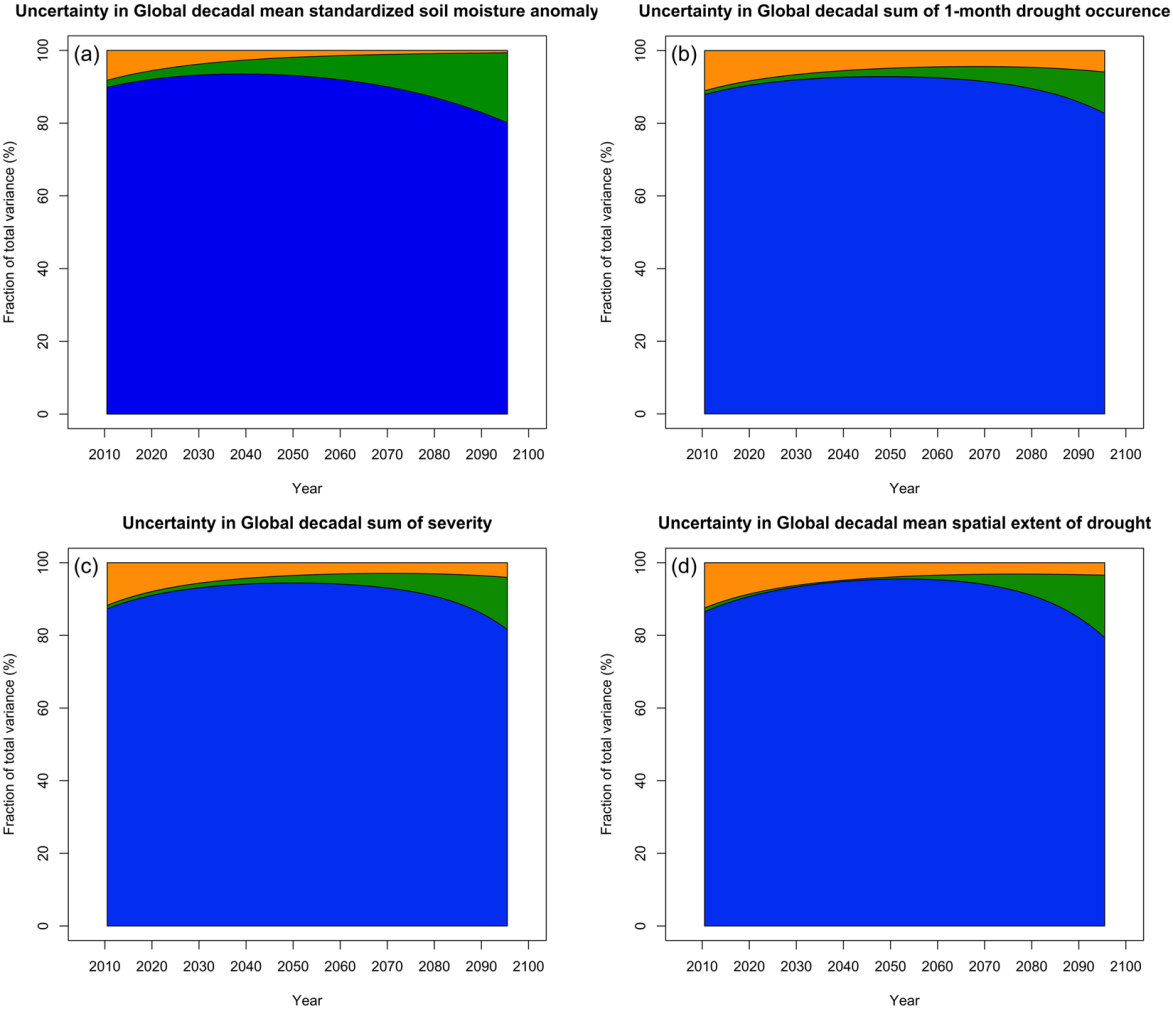
Fraction of total variance in (**a**) Global decadal mean standardized soil moisture anomaly, (**b**) Global decadal sum of 1-month drought occurrence, (**c**) Global decadal sum of severity, and (**d**) Global decadal mean spatial extent of drought, explained by three components of total uncertainty: internal variability (orange), scenario uncertainty (green), and model uncertainty (blue). The four uncertainty partitions correspond to the four sets of time series in Fig. 6.

In addition to visually presenting the uncertainty of future agricultural drought change, we partition and quantify the three dominant sources of uncertainty in those projections following the methods of Hawkins and Sutton^24^ and Hawkins and Sutton^26^. The projection of soil moisture shows large model uncertainties (Fig. 7(a)) during the entire 21st century owing to the simplified hydrological models of many CMIP5 climate models^5^, even if soil moisture is expressed in standardized anomaly format that already reduces the differences in soil moisture among models. The difference in the model response is the largest source of uncertainty (over 80%) over the entire 21st century. In the period before 2030, internal variability is the second largest source of uncertainty. After 2030, the scenario uncertainty exceeds internal variability and becomes the second largest source of uncertainty.

The contributions to total uncertainty for the three drought statistics: 1-month drought occurrence, sum of severity, and spatial extent, all show similar patterns (Fig. 7(b–d)). The model uncertainty is always the dominant source of uncertainty during the entire 21st century. In the early period, the internal variability typically is the second largest source of uncertainty in the early half of the 21st century. By the latter half of the century, it is often exceeded by scenario uncertainty. Our finding that uncertainty in soil moisture projections is dominated by model differences contrasts with the uncertainty partition for global decadal annual mean temperature changes found by Hawkins and Sutton^24^. In the case of global temperature, model uncertainty is relatively high in the early part of the century, but steadily falls and is exceeded by scenario uncertainty by the middle of 21st century. By the end, the scenario uncertainty accounts for approximately 82% of the total uncertainty and the model uncertainty accounts for 18%. Our results for soil moisture more closely approach the uncertainty in precipitation projections observed by Hawkins and Sutton^26^ wherein model uncertainty is the largest source of uncertainty over the entire 21st century. Of course, differences in modeled precipitation contribute greatly to simulated soil moisture differences. Yet, it is revealing that scenario uncertainty is so diminished despite the important role of temperature on evapotranspiration rates. Undoubtedly, uncertainty in simulated soil moisture values also results from the complexity of the water balance system and the model treatment of important factors, such as land use/cover, soil characteristics, landforms, vegetation, and evapotranspiration.

Regional patterns of uncertainty partition in decadal mean spatial extent of severe drought are similar and model uncertainty dominant uncertainty (approximately 80%) for all regions in the 21st century (see Supplementary Fig. S3). The difference across regions results mainly from the slight differences in the magnitude of scenario uncertainty shown in Fig. 3.

### Signal to noise ratio analysis

**Figure 8:**
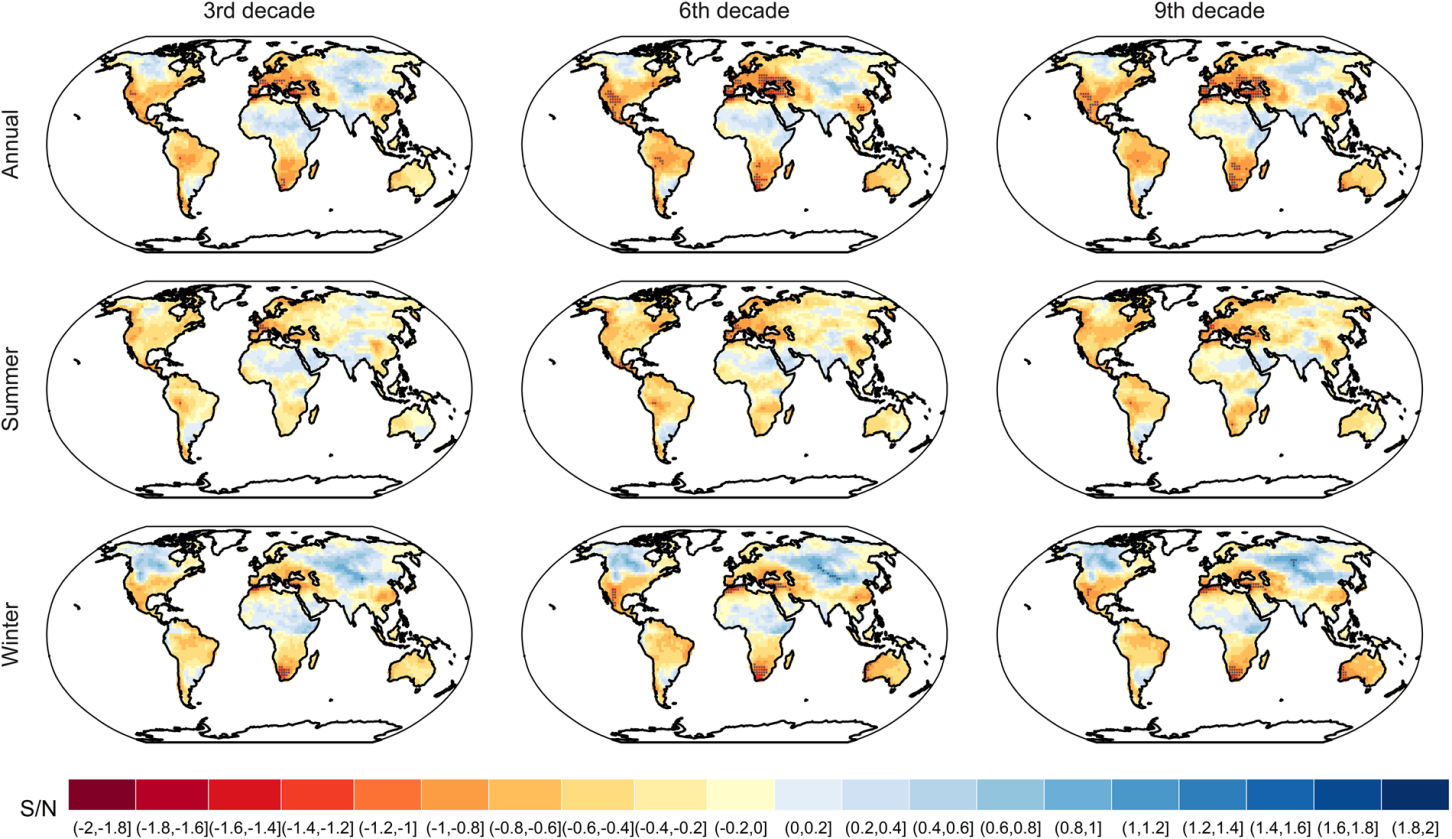
The annual and seasonal signal to noise ratio of surface soil moisture anomalies for the 3rd decade, 6th decade, and 9th decade relative to mean of 1976–2005 based on CMIP5 MMEs (summer: JJA in North hemisphere and DJF in South hemisphere; winter: DJF in North hemisphere and JJA in South hemisphere). The negative values indicate drying and the positive values indicate wetting. The grids with stippling indicate an absolute value of S/N greater than 1, which means that the magnitude of soil moisture anomaly change signal exceeds uncertainty.

We use the signal-to-noise ratio to measure how large the expected change of drought is compared to the uncertainty in the projections of drought. Understanding and modeling signal-to-noise ratio (S/N) for different regions could aid water resource planning. We quantify the signal as the change of soil moisture anomalies relative to the mean of the baseline period 1976–2005 and the noise as the square root of the total uncertainty (sum of internal variability, model uncertainty, and scenario uncertainty) in the projection^26,33^. We calculate the S/N ratio for each pixel. We also examine the spatiotemporal variability of annual and seasonal S/N change across the globe and at different lead times (3rd decade, 6th decade, and 9th decade) (Fig. 8). An absolute value of S/N greater than 1 means that the magnitude of soil moisture anomaly change signal exceeds uncertainty.

By the end of the 21st century, the annual negative S/N occurs across the Mediterranean and Europe, in many parts in United States, Mexico, southern Africa, many parts of northern South America, Southeast China, and West Australia. However, the S/N is less than −1 in a very limited region of the Mediterranean and Europe, Southwest United States, and southern Africa. The S/N ratio for drying is stronger in the summer than the winter. The annual positive S/N are found in limited regions including mid- to high-latitude Asia, high-latitude North America, India, east Argentina, and Sahara, but no regions show S/N values greater than 1. Positive S/N are more commonly found in the winter than the summer, especially in mid- to high-latitude Asia, and high-latitude North America. The spatial patterns of S/N do not change too much through time; while the magnitude of S/N becomes slightly greater, it does not change significantly. This indicates that the spread of uncertainty becomes larger as the signal becomes stronger.

We compare the S/N of soil moisture anomaly with the S/N of temperature and precipitation projections^26^. The S/N of temperature is far higher than that of soil moisture anomalies over all regions and all three lead times. The S/N of temperature peaks at the middle of the 21st century which is greater than 3 in the lower- to mid-latitude and even 4 in the tropics^26^. For precipitation, the highest wetting S/N is mainly shown in the high-latitude region. Over lead time, the high-latitude S/N of precipitation is increasing approximately from 1 to 2 and even larger than 2 but smaller than 3^26^. The drying S/N is mainly shown in the Mediterranean and Central America and the absolute value of S/N is increasing slightly from below 1 to above 1 over lead time^26^. Thus, the S/N of both temperature and precipitation are stronger than that of the soil moisture anomalies, with the S/N of temperature far stronger.

## Discussion and Conclusion

We have analyzed the raw surface soil moisture outputs from all available GCMs for four RCP scenarios: RCP2.6, RCP4.5, RCP6.0, and RCP8.5 under the CMIP5 framework. We evaluate the statistical significance of change in surface soil moisture using a Wilcoxon signed-rank test for each grid and control for false discovery rate (FDR) at a significance level of 0.05. We have found statistically significant annual drying over most of Australia, South America, North America, south Africa, Europe and Mediterranean, and east Asia, with stronger drying as the strength of forcing increases, but statistically significant wetting in limited areas of east Africa, south Asia, and central Asia by the end of the 21st century. The soil moisture drying in the Mediterranean, southwestern USA, northeast South America, and southern African is mainly associated with the projected widening of the Hadley Circulation and increased temperature and evapotranspiration^5^. The drying or wetting signal is more pronounced for seasonal than annual because of compensating effects for the whole year. We also have detected a strong seasonality in many regions in the mid- and high-latitude of North Hemisphere, with wetting in winter and drying in summer.

We calculate the duration, frequency, severity, and spatial extent of the severe agricultural drought (i.e., that which occurs approximately once every ten years for specific month). The multi-model median frequency of short-term drought is projected to increase by the end of the 21st century in most regions and most scenarios. In most cases, the increase in the frequency of short-term drought is higher for the RCP2.6 than the RCP8.5. In the latter scenario, individual months are more likely to cluster into consecutive dry months to form a long-term drought. The median frequency of long-term drought is also projected to increase in most regions, with the strongest increase in EUM, TSA, CAM, ENA, and SAF. The multi-model mean projects increased spatial extent of severe drought for all regions and all emission scenarios, with progressively larger spatial extent of severe drought as the strength of radiative forcing increases by the end of the 21st century. The multi-model and multi-scenario mean spatial extent of severe drought in CAM, EUM, TSA, and SAF are projected to increase the most. The increase in the spatial extent of drought tends to be larger in warmer seasons than cooler seasons in most regions because of increasing temperature and evapotranspiration. The inter-model variability is high and contributes the most to uncertainty in future projections and this source of uncertainty increases with radiative forcing, i.e., the model uncertainty is higher for RCP8.5 than for RCP2.6. Compared with the historical period (1976–2005), each individual GCM projection in the future (2071–2100) shows greater temporal (inter-month) variability in spatial extent of severe drought, indicating more widespread and extreme drought events. Furthermore, the variation in the spatial extent of drought across models are much larger than the variations across the RCP scenarios, i.e., the model spread is much larger than the scenario spread. The variation across models are also much larger than the variations across different regions, i.e., the model spread is much larger than the spatial heterogeneity.

In addition to visualizing the uncertainties, we have partitioned and quantified the three dominant sources of uncertainty with respect to decadal mean standardized soil moisture anomalies, decadal sum of 1-month drought occurrence, decadal sum of severity, and decadal mean spatial extent of drought from CMIP5 MMEs. We have found that more than 80% of the uncertainty associated with future drought projection in the 21st century comes from differences between GCMs, model uncertainty. This dominance results because of different treatment of clouds and precipitation in the models, as well as various local factors considered in soil moisture modeling. Regional patterns of uncertainty partition in spatial extent of drought for those 15 regions are similar to that for global and model uncertainty contributes approximately 80% of the total uncertainty. The differences across regions in uncertainty partition result mainly from the slight differences in the magnitude of scenario uncertainty.

When measured by simulated soil moisture conditions, drying occurs in large parts of the Mediterranean and Europe, many parts of the United States, Mexico, Southern Africa, many parts in Northern South America, Southeast China, West Australia, and the drying signal become stronger over lead time. However, since the inter-model variability is so high, the S/N of drying in soil moisture is less than −1 in only limited regions including the Mediterranean and Europe, the southwestern United States, and southern Africa. The wetting occurs in mid- to high-latitude Asia, high-latitude North America, India, east Argentina, and Sahara, but none of these regions have an S/N ratio greater than 1. The spread of uncertainty becomes larger as the signal becomes stronger and thus the spatial pattern and magnitude of S/N does not change significantly.

Improving future projection of agricultural drought depends on improved model performance in simulating soil moisture, e.g., improved representation of the surface hydrological process. The GCMs might have limited ability to simulate the water cycle and all relevant interactions between the atmosphere and land surface^5^. This situation is further complicated by the fact of error propagation that model biases in one variable affect other variables through the causal chain (e.g. the simulation of soil moisture depends on the simulation of precipitation and evapotranspiration, representation of the soil layers and soil characteristics, etc.). The model uncertainty could be attributed to imperfect representation of the processes, or limited understanding of the very complex process, or inherent challenges in mathematically representing the processes^5^, e.g., previous study also pointed out that there are large differences among CMIP5 GCMs to represent the permafrost soil thermal dynamics and the coupling between soil and air temperatures in the high latitude resulting in difference in snow physics and soil hydrology^34^. Additionally, this study uses the upper 10 cm surface soil moisture, instead of the total soil moisture. The surface soil moisture (upper 10 cm) data for different models are quantitatively more comparable than the total soil moisture due to the substantial differences between climate models in the soil depth and soil layers^5,35^. The total soil moisture should have more uncertainty than the surface soil moisture because of differences in soil depth and layers. Many vegetation types are able to extract water from deeper soil layers^35,36^ and thus we need soil moisture of deeper layers with consistent soil depth and layers across different GCMs to better quantify the agricultural drought. Thus, we also advocate to standardize the soil schemes and improve the representation of soil layers across different GCMs from different modeling centers to reduce this structural uncertainty due to differences in soil depth and layers.

## Methods

### Climate models

We used climate model simulations under the framework of CMIP5^5,37–39^. Four emissions scenarios, called representative concentration pathways (RCPs) were used. Each is identified by its approximate total radiative forcing W/m^2^ in year 2100 relative to preindustrial conditions (1750): RCP2.6, RCP4.5, RCP6.0 RCP8.5^5^. The radiative forcing of RCP2.6 peaks first and then declines, representing the lowest scenario; the radiative forcing of RCP4.5 stabilizes at 4.5 W/m^2^ by 2100, representing the medium-low scenario; the radiative forcing of RCP6.0 and RCP8.5 does not stabilize by 2100, representing the medium-high and highest scenario respectively. The CMIP5 multi-model ensembles (MMEs) are available via portals to the Earth System Grid Federation (ESGF) archive (http://cmip-pcmdi.llnl.gov/cmip5/).

We used the monthly surface (upper 10 cm) soil moisture (variable: mrsos) outputs from CMIP5 MMEs for historical simulations (1900–2005) and future projections (2006–2100). We used all available models providing surface soil moisture values during the simulation periods (listed in Supplementary Table S1). To enable comparison across the four RCP scenarios, Figs 2, 3 and 5 only contain models that are available across all RCP scenarios and historical forcing (those models are annotated with star symbols in Supplementary Table S1). All model outputs were interpolated onto a common 2° × 2° latitude-longitude grid by bilinear interpolation method to allow for computing multi-model mean and uncertainty. The multi-ensemble mean was calculated for each GCM and each scenario.

### Soil moisture anomalies

Near-surface soil moisture is a function of precipitation, evapotranspiration, soil texture and infiltration, drainage, slope, vegetation cover, etc. which are heterogeneous and difficult to characterize^5^. The surface soil moisture (upper 10 cm) provided by CMIP5 differs greatly model by model. For example, the global (excluding Antarctica and Greenland) annual mean surface soil moisture for the period of 1976–2005 varies from 8.381 kg/m^2^ in model IPSL-CM5A-MR to 33.598 kg/m^2^ in model FGOALS-s2, while the standard deviation for the period of 1976–2005 is only 0.058 kg/m^2^ in model IPSL-CM5A-MR and 0.089 kg/m^2^ in model FGOALS-s2. Differences between models are far greater than interannual variability for a single model. Thus, comparing the raw surface soil moisture between models is not robust. Here, we compute the soil moisture anomalies using future projections (or historical simulation) minus the mean of historical simulation (1900–2005). In addition, since the interannual variability of soil moisture differs by model, we also compute the standardized soil moisture anomalies to investigate how future long-term changes compare to historical interannual variability^40^. The standardized soil moisture anomalies are computed using future projections (or historical simulation) minus the mean of historical simulation (1900–2005) and normalized by interannual standard deviation of historical simulation (1900–2005). The anomalies are calculated for each model due to differences model by model, calculated for each month due to varying soil moisture conditions each month, and calculated for each pixel due to spatial heterogeneity.

### Drought quantification

We estimate future wetting and drying of surface soil moisture with respect to the historical empirical probability distribution^23^. Our approach considers United States Drought Monitor (USDM) drought classification categories: D0 abnormally dry (21st to 30th percentile), D1 moderate drought (11st to 20th), D2 severe drought (6th to 10th), D3 extreme drought (3rd to 5th), and D4 exceptional drought (0 to 2nd) (http://droughtmonitor.unl.edu/). Here, we focus on severe drought and even worse (0 to 10th percentile), i.e., including D2, D3, and D4 drought category in USDM. A threshold value of 10% is chosen reflecting a drought condition for a specific month that could be expected once every ten years on average. Hence, following the method of Sheffield and Wood^23^, for each grid point, each month, and each GCM, an empirical cumulative distribution function (ECDF) is calculated for the surface soil moisture from the historical simulation (1900–2005) and then the 10% quantile threshold of surface soil moisture is computed. A 1-month drought occurrence either in the historical period or future period is defined as the month with a surface soil moisture value lower than the 10% quantile threshold based on the ECDF. We selected a long enough historical period (106-year, 1900–2005) to sufficiently account for the historical variability in surface soil moisture.

Based on the theory of runs and the method of Sheffield and Wood^23^, a drought event is characterized in terms of duration, severity, intensity, and spatial extent. A consecutive sequence of 1-month drought occurrence results in a drought event of different durations. We define two types of drought duration based on the USDM: short-term drought (less than 6 months) and long-term drought (longer than or equal to 6 months). We define severity as the sum of deficit below the 10% threshold. For example, the cumulative probability of the surface soil moisture in the 1st, 2nd, and 3rd month during a 3-month drought event are respectively 6%, 2%, and 3%, and hence the deficit below the 10% threshold for those three months is 4%, 8%, and 7% respectively. Consequently, the sum of deficit below the threshold is 19%, i.e., the severity of this drought event is 19%. We define intensity as the mean deficit below the threshold for a drought event, i.e., severity divided by duration. For example, the intensity of this 3-month duration drought mentioned above is 19%/3 (6.33%). We define spatial extent of drought as the percentage of grid points in which the surface soil moisture falls below the threshold for each month in the region of interest. The area of each grid point is weighted by the cosine of the latitude to account for the actual grid size. We estimate the drought characteristics for the 15 regions defined by the IPCC^5^ (Fig. 2(b)) and calculate the regional mean for each drought statistic using an area-weighted mean by the cosine of the latitudes.

### Uncertainty quantification and partition

Future climate change projections are subject to considerable uncertainties. Here, we consider three dominant sources of uncertainty in drought projection^24^: (1) internal variability of climate system, i.e., natural fluctuation, which arises in the absence of any radiative forcing; (2) model uncertainty (known as response uncertainty), which occurs because different GCMs project different climate changes in response to the same radiative forcing; and (3) scenario uncertainty, which arises from uncertainty in future anthropogenic greenhouse gas emissions, leading to uncertainty in future radiative forcing.

We follow the methods in Hawkins and Sutton^24,26^ to partition and quantify the uncertainties for drought projection. Here, we describe the method in brief. (1) For each individual projection, we fit a smooth fourth-order polynomial model to the decadal mean projection during the period, 1900–2100, to account for the non-linearity and to separate out the trend and internal variability. Each model is assumed independent for simplicity, although they don’t (Fig. 5). The internal variability for each projection is defined as the variance of the residuals from the smooth fit. We assume that the internal variability is constant over lead time and that changes of internal variability are negligible. We take the multi-model mean of the variances of the residuals as the internal variability component. (2) For one particular scenario, the spread of different models is considered as the model uncertainty. We estimate the model uncertainty for each scenario as the variance of the smooth fits for different models. The multi-scenario mean of the variance is considered as an estimate of model uncertainty. (3) The spread of the multi-model mean for each scenario is considered as the scenario uncertainty. We estimate the scenario uncertainty as the variance of the multi-model means for the four scenarios. The model uncertainty and scenario uncertainty varies by lead time. Those three uncertainties are assumed independent from one another^24^. The total uncertainty is estimated as the sum of internal variability, model uncertainty, and scenario uncertainty.

## Supplementary information

Supplementary Information

## References

[1] AMS. Drought — An Information Statement of the American Meteorological Society, https://www.ametsoc.org/ams/index.cfm/about-ams/ams-statements/statements-of-the-ams-in-force/drought/ (2013).

[2] IPCC. Climate Change 2007: The Physical Science Basis. Contribution of Working Group I to the Fourth Assessment Report of the Intergovernmental Panel on Climate Change (Cambridge University Press, 2007).

[3] Mishra AK, Singh VP (2011). Drought modeling - A review. J Hydrol.

[4] Heinrich G, Gobiet A (2012). The future of dry and wet spells in Europe: a comprehensive study based on the ENSEMBLES regional climate models. Int. J. Climatol..

[5] IPCC. Climate Change 2013: The Physical Science Basis. Contribution of Working Group I to the Fifth Assessment Report of the Intergovernmental Panel on Climate Change. (Cambridge University Press, 2013).

[6] Sheffield J, Wood EF, Roderick ML (2012). Little change in global drought over the past 60 years. Nature.

[7] Dai A (2013). Increasing drought under global warming in observations and models. Nature Clim. Change.

[8] Loukas A, Vasiliades L, Tzabiras J (2008). Climate change effects on drought severity. Adv. Geosci.

[9] Vidal J-P, Wade S (2009). A multimodel assessment of future climatological droughts in the United Kingdom. Int. J. Climatol..

[10] Mishra, A. K. & Singh, V. P. Analysis of drought severity-area-frequency curves using a general circulation model and scenario uncertainty. *J Geophys Res-Atmos* **114**, D06120, 10.1029/2008jd010986 (2009).

[11] Dubrovsky M (2009). Application of relative drought indices in assessing climate-change impacts on drought conditions in Czechia. Theoretical and Applied Climatology.

[12] Touma D, Ashfaq M, Nayak MA, Kao S-C, Diffenbaugh NS (2015). A multi-model and multi-index evaluation of drought characteristics in the 21st century. J Hydrol.

[13] Palmer, W. C. Meteorological drought. Vol. 30 (US Department of Commerce, Weather Bureau Washington, DC, USA, 1965).

[14] Vicente-Serrano SM, Beguería S, López-Moreno JI (2010). A Multiscalar Drought Index Sensitive to Global Warming: The Standardized Precipitation Evapotranspiration Index. J Climate.

[15] Rind D, Goldberg R, Hansen J, Rosenzweig C, Ruedy R (1990). Potential Evapotranspiration and the Likelihood of Future Drought. J Geophys Res-Atmos.

[16] McKee, T. B., Doesken, N. J. & Kleist, J. The relationship of drought frequency and duration to time scales. In *Proceedings of the 8th Conference on Applied Climatology* **17**, 179–183 (1993).

[17] Shukla, S. & Wood, A. W. Use of a standardized runoff index for characterizing hydrologic drought. *Geophys. Res. Lett*. **35**, L02405, 10.1029/2007GL032487 (2008).

[18] AMS (1997). Meteorological drought — Policy statement. B Am Meteorol Soc.

[19] Heim RR (2002). A review of twentieth-century drought indices used in the United States. B Am Meteorol Soc.

[20] Lu J, Carbone GJ, Gao P (2017). Detrending crop yield data for spatial visualization of drought impacts in the United States, 1895–2014. Agr Forest Meteorol.

[21] WMO. Drought and Agriculture. *WMO Technical Note 138*. 127 (1975).

[22] Keyantash J, Dracup JA (2002). The quantification of drought: An evaluation of drought indices. B Am Meteorol Soc.

[23] Sheffield J, Wood EF (2008). Projected changes in drought occurrence under future global warming from multi-model, multi-scenario, IPCC AR4 simulations. Clim Dynam.

[24] Hawkins E, Sutton R (2009). The Potential to Narrow Uncertainty in Regional Climate Predictions. B Am Meteorol Soc.

[25] Morice, C. P., Kennedy, J. J., Rayner, N. A. & Jones, P. D. Quantifying uncertainties in global and regional temperature change using an ensemble of observational estimates: The HadCRUT4 data set. *J Geophys Res-Atmos* **117**, D08101, 10.1029/2011JD017187 (2012).

[26] Hawkins E, Sutton R (2011). The potential to narrow uncertainty in projections of regional precipitation change. Clim Dynam.

[27] Rowell DP (2012). Sources of uncertainty in future changes in local precipitation. Clim Dynam.

[28] Yuan S, Quiring SM (2017). Evaluation of soil moisture in CMIP5 simulations over the contiguous United States using *in situ* and satellite observations. Hydrol. Earth Syst. Sci..

[29] Stillman S, Zeng X, Bosilovich MG (2015). Evaluation of 22 Precipitation and 23 Soil Moisture Products over a Semiarid Area in Southeastern Arizona. Journal of Hydrometeorology.

[30] Ruosteenoja K, Markkanen T, Venäläinen A, Räisänen P, Peltola H (2018). Seasonal soil moisture and drought occurrence in Europe in CMIP5 projections for the 21st century. Clim Dynam.

[31] Mishra V, Shah R, Thrasher B (2014). Soil Moisture Droughts under the Retrospective and Projected Climate in India. Journal of Hydrometeorology.

[32] Benjamini Y, Hochberg Y (1995). Controlling the False Discovery Rate: A Practical and Powerful Approach to Multiple Testing. Journal of the Royal Statistical Society. Series B (Methodological).

[33] Giorgi, F. & Bi, X. Time of emergence (TOE) of GHG-forced precipitation change hot-spots. *Geophys. Res. Lett*. **36**, L06709, 10.1029/2009GL037593 (2009).

[34] Koven CD, Riley WJ, Stern A (2012). Analysis of Permafrost Thermal Dynamics and Response to Climate Change in the CMIP5 Earth System Models. J Climate.

[35] Berg A, Sheffield J, Milly PCD (2017). Divergent surface and total soil moisture projections under global warming. Geophys. Res. Lett..

[36] Canadell J (1996). Maximum rooting depth of vegetation types at the global scale. Oecologia.

[37] Hurrell, J., Visbeck, M. & Pirani, P. WCRP Coupled Model Intercomparison Project-Phase 5-CMIP5. *Clivar Exchanges* **16** (2011).

[38] Taylor KE, Stouffer RJ, Meehl GA (2012). An Overview of CMIP5 and the Experiment Design. B Am Meteorol Soc.

[39] Meehl GA (2009). Decadal Prediction. B Am Meteorol Soc.

[40] Koster RD (2009). On the Nature of Soil Moisture in Land Surface Models. J Climate.

