# Supplementary material for: Uncertainty and hotspots in 21st century projections of agricultural drought from CMIP5 models
Source: Sci Rep. 2019 Mar 20;9:4922. doi: 10.1038/s41598-019-41196-z (PMC6426967; doi:10.1038/s41598-019-41196-z)
Supplement: Supplementary file 1 — Supplementary Information [file 41598_2019_41196_MOESM1_ESM.docx]

Uncertainty and hotspots in 21st century projections of agricultural drought from CMIP5 models

Junyu Lu^1,*^, Gregory J. Carbone^1^, John M. Grego^2^

1 Department of Geography, University of South Carolina, Columbia, South Carolina 29208, United States

2 Department of Statistics, University of South Carolina, Columbia, South Carolina 29208, United States

* Corresponding author: jlu@email.sc.edu

**Supplementary Information**

Table S1 List of GCMs and number of ensembles for each GCM and each scenario (the blanks indicate no simulation available and the numbers indicate the number of ensembles used and the model name with a star symbol indicate this model has all four RCP scenario runs, totally 17)

| Model Center | Institution | Model | Historical | RCP2.6 | RCP4.5 | RCP6.0 | RCP8.5 |
| --- | --- | --- | --- | --- | --- | --- | --- |
| CSIRO-BOM | CSIRO (Commonwealth Scientific and Industrial Research Organisation, Australia), and BOM (Bureau of Meteorology, Australia) | ACCESS1.0 | 3 |  | 1 |  | 1 |
|  |  | ACCESS1.3 | 3 |  | 1 |  | 1 |
| BCC | Beijing Climate Center, China Meteorological Administration, China | BCC-CSM1.1 | 3 | 1 | 1 |  | 1 |
|  |  | BCC-CSM1.1(m) * | 3 | 1 | 1 | 1 | 1 |
| GCESS | College of Global Change and Earth System Science, Beijing Normal University | BNU-ESM | 1 | 1 | 1 |  | 1 |
| CCCma | Canadian Centre for Climate Modelling and Analysis | CanESM2 | 5 | 5 | 5 |  | 5 |
| NSF-DOE-NCAR | National Science Foundation, Department of Energy, National Center for Atmospheric Research | CESM1(BGC) | 1 |  | 1 |  | 1 |
|  |  | CESM1(CAM5) * | 3 | 3 | 3 | 3 | 3 |
| CSIRO-QCCCE | Commonwealth Scientific and Industrial Research Organisation in collaboration with the Queensland Climate Change Centre of Excellence | CSIRO-Mk3.6.0 * | 10 | 10 | 10 | 10 | 10 |
| LASG-CESS | LASG, Institute of Atmospheric Physics, Chinese Academy of Sciences; and CESS, Tsinghua University | FGOALS-g2 | 5 | 1 | 1 |  | 1 |
| LASG-IAP | LASG, Institute of Atmospheric Physics, Chinese Academy of Sciences | FGOALS-s2 | 3 | 1 |  | 1 | 3 |
| NOAA GFDL | Geophysical Fluid Dynamics Laboratory | GFDL-CM3 * | 4 | 1 | 1 | 1 | 1 |
|  |  | GFDL-ESM2G * | 1 | 1 | 1 | 1 | 1 |
|  |  | GFDL-ESM2M * | 1 | 1 | 1 | 1 | 1 |
| NASA-GISS | NASA Goddard Institute for Space Studies | GISS-E2-H * | 18 | 3 | 15 | 3 | 5 |
|  |  | GISS-E2-H-CC | 1 |  | 1 |  | 1 |
|  |  | GISS-E2-R * | 26 | 3 | 17 | 3 | 5 |
|  |  | GISS-E2-R-CC | 1 |  | 1 |  | 1 |
| MOHC | Met Office Hadley Centre (additional HadGEM2-ES realizations contributed by Instituto Nacional de Pesquisas Espaciais) | HadGEM2-ES * | 3 | 4 | 4 | 2 | 4 |
| INM | Institute for Numerical Mathematics | INM-CM4 | 1 |  | 1 |  | 1 |
| IPSL | Institut Pierre-Simon Laplace | IPSL-CM5A-LR * | 6 | 4 | 4 | 1 | 4 |
|  |  | IPSL-CM5A-MR * | 3 | 1 | 1 | 1 | 1 |
|  |  | IPSL-CM5B-LR | 1 |  | 1 |  | 1 |
| MIROC | Japan Agency for Marine-Earth Science and Technology, Atmosphere and Ocean Research Institute (The University of Tokyo), and National Institute for Environmental Studies | MIROC-ESM * | 3 | 1 | 1 | 1 | 1 |
|  |  | MIROC-ESM-CHEM * | 1 | 1 | 1 | 1 | 1 |
| MIROC | Atmosphere and Ocean Research Institute (The University of Tokyo), National Institute for Environmental Studies, and Japan Agency for Marine-Earth Science and Technology | MIROC5 * | 4 | 3 | 3 | 3 | 3 |
| MRI | Meteorological Research Institute | MRI-CGCM3 * | 5 | 1 | 1 | 1 | 1 |
|  |  | MRI-ESM1 | 1 |  |  |  | 1 |
| NCC | Norwegian Climate Centre | NorESM1-M * | 3 | 1 | 1 | 1 | 1 |
|  |  | NorESM1-ME * | 1 | 1 | 1 | 1 | 1 |





Figure S1. Same as Figure 1 with summer (JJA in North hemisphere and DJF in South hemisphere)





Figure S2. Same as Figure 1 with winter (DJF in North hemisphere and JJA in South hemisphere)


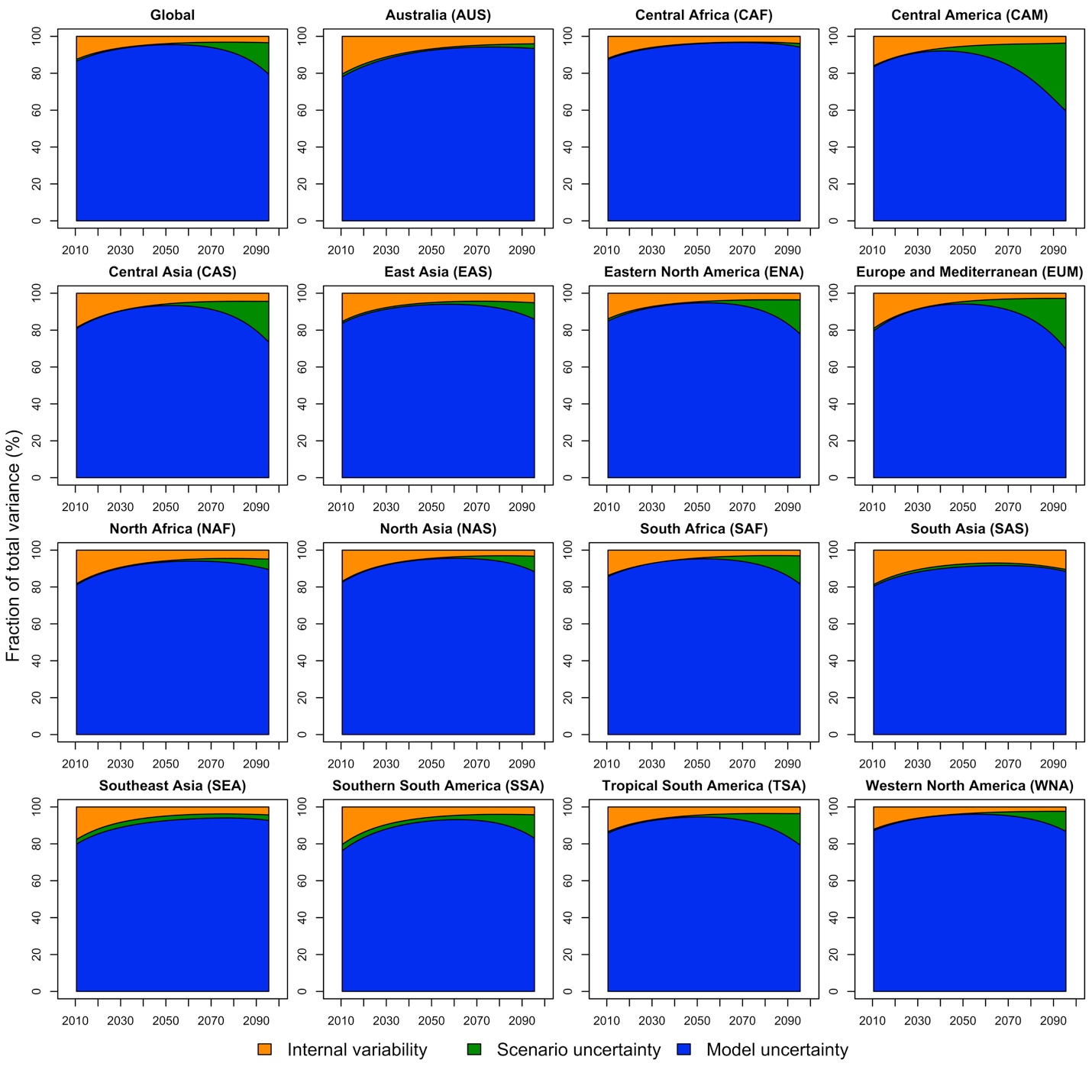


Figure S3. Fraction of total variance in global and regional decadal mean spatial extent of drought, explained by three components of total uncertainty: internal variability (orange), scenario uncertainty (green), and model uncertainty (blue).
